# When and Why Adults Abandon Lifestyle Behavior and Mental Health Mobile Apps: Scoping Review

**DOI:** 10.2196/56897

**Published:** 2024-12-18

**Authors:** Patrick G Kidman, Rachel G Curtis, Amanda Watson, Carol A Maher

**Affiliations:** 1 Alliance for Research in Exercise, Nutrition and Activity Allied Health and Human Performance University of South Australia Adelaide Australia; 2 Flinders Health and Medical Research Institute College of Medicine and Public Health Flinders University Adelaide Australia

**Keywords:** mobile health apps, smartphone applications, app abandonment, app attrition, user engagement, health behavior, user retention, lifestyle management, quantitative analysis, qualitative analysis, mobile phone

## Abstract

**Background:**

With 1 in 3 adults globally living with chronic conditions and the rise in smartphone ownership, mobile health apps have become a prominent tool for managing lifestyle-related health behaviors and mental health. However, high rates of app abandonment pose challenges to their effectiveness.

**Objective:**

We explored the abandonment of apps used for managing physical activity, diet, alcohol, smoking, and mental health in free-living conditions, examining the duration of app use before abandonment and the underlying reasons.

**Methods:**

A scoping review was conducted based on the PRISMA-ScR (Preferred Reporting Items for Systematic reviews and Meta-Analyses extension for Scoping Reviews) guidelines and eligibility criteria were designed according to the SPIDER (Sample, Phenomenon of Interest, Design, Evaluation, Research type) framework. In total, 4 databases were searched (MEDLINE, Scopus, Embase, and PsycINFO) to identify quantitative and qualitative studies with outcome measures related to app abandonment in adults with free-living conditions, including reasons for abandonment and duration of use, for mobile apps related to WHO (World Health Organization) modifiable health behaviors and mental health. The included studies’ risk of bias was appraised based on the STROBE (Strengthening the Reporting of Observational Studies in Epidemiology) and COREQ (Consolidated Criteria for Reporting Qualitative Research) checklists. To enable data synthesis across different methodologies, app domains, demographic data, and outcome measures were categorized. Results are presented in 2 sections: quantitatively in a scatterplot to understand when users abandon apps and qualitatively through basic qualitative content analysis to identify the underlying reasons.

**Results:**

Eighteen eligible studies (525,824 participants) published between 2014 and 2022, predominantly from the United States, Canada, the United Kingdom, and Germany, were identified. Findings revealed a curvilinear pattern of app abandonment, with sharper abandonment soon after acquisition, followed by a slowing rate of abandonment over time. Taken together, a median of 70% of users discontinued use within the first 100 days. The abandonment rate appeared to vary by app domain, with apps focusing on alcohol and smoking exhibiting faster abandonment, and physical activity and mental health exhibiting longer usage durations. In total, 22 unique reasons for abandonment were organized into six categories: (1) technical and functional issues, (2) privacy concerns, (3) poor user experience, (4) content and features, (5) time and financial costs, and (6) evolving user needs and goals.

**Conclusions:**

This study highlights the complex nature of health app abandonment and the need for an improved understanding of user engagement over time, underscoring the importance of addressing various factors contributing to abandonment, from technical issues to evolving user needs. Our findings also emphasize the need for longitudinal studies and a consistent definition of app abandonment to better understand and mitigate this phenomenon, thereby enhancing the effectiveness of health apps in supporting public health initiatives.

## Introduction

A total of 1 in 3 adults worldwide live with multiple chronic conditions [1], a situation exacerbated by modifiable health behaviors such as tobacco use, alcohol consumption, diet, sleep patterns, physical activity, and sedentary habits [2]. Furthermore, mental illnesses such as depression and anxiety are significant sources of global disability [3]. Mobile health apps offer a promising avenue for enhancing both physical [4] and mental health outcomes [5] by facilitating improvements in these health behaviors.

As of 2023, an estimated 6.7 billion people around the world own smartphones [6]. In 2022, the global mobile health market was valued at US $50 billion and is projected to grow to US $466 billion by 2032 [7]. The health and fitness app market is substantial, with a reported 350,000 health apps available on the Apple and Google Play Stores, and around 90,000 new health apps added in 2020 alone (an average of 250 per day) [8]. Despite their undoubted potential, industry data shows that 66% of health apps and 69% of fitness apps are abandoned within 90 days, which is worse than the average of 52% for all app categories [9]. This represents a tremendous waste of time and resources, particularly given that, on average, development takes 7-12 months and costs US $270,000 [10].

Numerous studies have examined health app adoption and usage [11-14], suggesting that individuals select apps based on specific health needs and preferences. A desire to improve health, through health behaviors like diet [12] or physical activity [15], is a common driver. App reviews and ratings, the credibility of the source, and recommendations from health care practitioners, friends, or family members have all been identified as important determinants of app adoption [13]. Specific features, such as the ability to track outcomes over time, data visualization, and gamification elements also play a role [11]. Cost and accessibility also impact the choice of health apps, with free apps generally favored, although subscription models can be successful if they are perceived as offering value [11]. Two 2022 systematic reviews, by Jakob et al [16], and Amagai et al [14] examined the factors influencing health app adherence and engagement. They identified a total of 99 and 62 previous studies, respectively, which, taken together, suggested that user-friendliness, tailoring, reminders, in-app support from health care professionals, gamification, and financial incentives are key factors in improving app adherence. While these studies provide insight into reasons for the adoption and usage of health apps, less is known about app abandonment.

In 2020, Meyerowitz-Katz et al [17] published a systematic review specifically examining attrition in app-based intervention studies for chronic diseases. They identified 17 studies, including apps designed for a cast array of chronic diseases and management approaches, including HIV management, management of menstrual pain, low back pain, and chronic kidney disease, including intervention approaches such as pain management, medication adherence, and more generalized health behaviors such as healthy eating and physical activity. They noted that most studies have focused on reporting attrition rates quantitatively, without attempting to determine the reasons behind the attrition. They also reported that attrition rates appeared to vary depending on whether it was studied under randomized controlled trial conditions (lower dropout) or under “real-world” conditions (higher dropout). This fits with a wider commentary about the limited real-world applicability of results obtained under RCT conditions being reported across a range of health and medical research contexts [18,19].

This study set out to address these gaps. The objective of this review is to explore the abandonment of apps used for managing lifestyle behaviors (physical activity, diet, alcohol, and smoking) and mental health (henceforth referred to as “health apps” for simplicity), collected under free-living conditions. Furthermore, we will bring together abandonment data with qualitative research exploring the reasons why people abandon health apps. We hope that this will provide enhanced understanding to guide the design of health apps that are less likely to be abandoned in the future.

## Methods

### Protocol

This scoping review was conducted with reference to and reported according to the PRISMA-ScR (Preferred Reporting Items for Systematic Reviews and Meta-Analyses extension for Scoping Reviews; refer to Multimedia Appendix 1 for checklist) [20].

### Eligibility Criteria

The eligibility criteria (Table 1) were developed using the Sample, Phenomenon of Interest, Design, Evaluation, and Research type (SPIDER) framework [21].

**Table 1:** Eligibility criteria according to the Sample, Phenomenon of Interest, Design, Evaluation, and Research type framework.

SPIDER^a^	Inclusion	Exclusion
Sample	Adults (aged ≥18 years), smartphone users, general population with no restrictions on health status.	Pediatric samples, populations other than general smartphone users (eg, app developers).
Phenomenon of Interest	Mobile health apps related to the WHO^b^ modifiable health behaviors (physical activity, diet, weight loss, sedentary behavior, smoking, sleep, and alcohol use) [2] or mental health.	Mobile health apps focusing on managing chronic conditions (eg, tracking health symptoms such as glucose, blood pressure, or pain and managing medication), making medical appointments, or accessing telehealth services. Research involving the use of wearable technology, such as fitness trackers.
Design	All qualitative (eg, interviews, focus groups) and quantitative (eg, surveys, app use data) observational research designs in free-living conditions (ie, where participants used a publicly available app before enrolling in the research study).	Research in non-free-living conditions (eg, clinical trials, app development, and testing) and secondary research (reviews).
Evaluation	Outcome measures relating to app abandonment such as reasons for app abandonment (eg, technical issues, privacy) and duration of app use.	No app abandonment outcome measures are present.
Research type	Qualitative, quantitative, or mixed methods research designs. Peer-reviewed journal articles published in English from 2007 onwards to coincide with the first iPhone (Apple Inc) release. We focused on peer-reviewed literature to ensure a high standard of evidence quality and maintain feasibility within our resource constraints.	Research in non-free-living conditions. Gray literature and studies not published in English.

^a^SPIDER: Sample, Phenomenon of Interest, Design, Evaluation, Research type.

^b^WHO: World Health Organization.

### Information Sources and Search Strategy

A database search was conducted on December 5, 2022, and included 4 databases: MEDLINE, Scopus, Embase, and PsycINFO. The search strategy was devised with the academic team and finalized with the support of an academic librarian.

The searches consisted of combining synonyms for the phenomenon of interest (mobile health apps), app types (diet, physical activity, etc), and evaluation (app abandonment). The search was also restricted to adult humans, English, and studies from 2007 (when the first iPhone was released) to the present. Terms were mapped to MeSH (Medical Subject Headings) as appropriate. Reference lists of included studies were scanned, to determine additional studies that met the inclusion criteria. The full search strategy can be found in Multimedia Appendix 2.

### Evidence Selection and Data Charting

Database search results were collated and imported into EndNote V.x9 (Clarivate). Duplicates were removed in EndNote and checked manually. Studies were screened in Covidence (Veritas Health Innovation) based on title and abstracts in duplicate by 2 independent reviewers (PGK and AW), as per best practice to reduce bias [22]. The results of the screening were compared and discussed until a consensus was reached. Another independent reviewer (CAM and RGC) was consulted as needed, to assist in resolving any discrepancies.

Next, full-text screening was conducted to define eligible studies. This was conducted by 3 independent reviewers. PGK, RGC, and AW completed a pilot full-text screening of 5 studies to test the criteria. RGC and PGK completed 20% (13/63) of the full-text screening in duplicate, with a Cohen κ score of 0.82. PGK completed the screening of the remaining studies.

A custom document was generated and used for data charting (Multimedia Appendix 3). Charted data included country of origin, study design, health app domain, aims or objectives, sample size, population, app abandonment definition, and outcome measures (reasons for abandonment or duration). If unclear, the authors were contacted to confirm the data. Pilot data charting of 5 studies (28%, 5/18) was conducted to test the quality of the form. PGK completed data charting and, if there were any concerns, other members of the team were contacted to reach a unanimous verdict before proceeding.

### Critical Appraisal

Two tools were used to determine the risk of bias based on the STROBE (Strengthening the Reporting of Observational Studies in Epidemiology) [23] checklist for observational studies and the COREQ (Consolidated Criteria for Reporting Qualitative Research) [24] checklist for qualitative studies. These 2 tools were needed due to the heterogeneity of the methodologies of the included studies. Mixed methods studies were evaluated using the tool that captured the majority of charted data. If the study met the criteria, it received a 1. If the item was not applicable or not satisfied, it was scored 0. PGK completed data charting and risk of bias, consulting the team for advice in the case of uncertainty. While not required for scoping reviews, we conducted a critical appraisal to enhance the rigor of our review and provide context for interpreting findings. This aligns with PRISMA-ScR guidelines and helps identify methodological gaps in the field.

### Synthesis of Results

In order to facilitate data synthesis across diverse research designs, overarching categories were constructed to describe the health domains, study design, and outcomes using qualitative content analysis. After data extraction was completed, a list of all reported health domains and outcomes were compiled. After familiarization with the data, the first author sorted them into similar categories using an inductive approach (ie, directed by the data with no preconceived categories) [25]. These categories were reviewed with the senior author, refined, and named.

A PRISMA (Preferred Reporting Items for Systematic Reviews and Meta-Analyses) flowchart of the search and screening process was presented along with a descriptive discussion of these results. A study characteristics table used descriptive statistics (n and %) to describe the year, country, health app domain, outcome measures, and study design. The risk of bias for the included studies based on either the STROBE or COREQ reporting guidelines was reported descriptively.

Due to the review aims and study heterogeneity, the results were presented in 2 sections. First, quantitative findings were presented in a scatterplot depicting duration for use for different health app domains over time, before being discussed descriptively with the range of abandonment percentage intervention lengths calculated.

For the synthesis of qualitative data, a basic qualitative content analysis using an inductive approach was used, enabling the organization of categories and subcategories from the data [25,26]. To do this, codes were assigned to the extracted qualitative data on reasons for app abandonment. We then organized the codes into categories and subcategories, to reach an integrated understanding of why people abandoned apps. Coding was primarily undertaken by PGK and reviewed by the other 3 authors.

## Results

### Selection of Sources of Evidence

**Figure 1:**
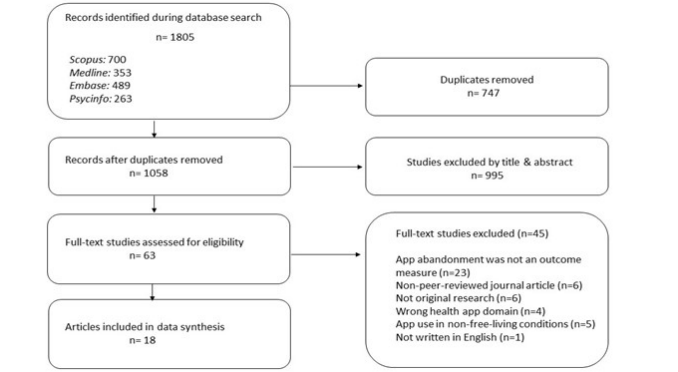
PRISMA (Preferred Reporting Items for Systematic reviews and Meta-Analyses) flowchart.

The database search discovered 1805 studies (see Figure 1 for PRISMA flow diagram), with 1058 unique studies sent to title and abstract screening after the removal of duplicates (n=747). In total, 63 studies were assessed during full-text screening, with 45 being excluded. Reasons included not having an outcome measure related to app abandonment (n=23), nonpeer-reviewed research (n=6), not original research (n=6), wrong health app domain (n=5), non–free-living conditions (n=4) and not being written in English (n=1). As a result, 18 studies were deemed eligible for this review [11,12,27-42].

### Characteristics for Sources of Evidence

All 18 studies [11,12,27-42] included in this review were written in English and published between 2014 and 2022, with half being published between 2019 and 2022. The total sample size was 525,824 participants, with the range being 10-189,770 participants. Most studies were conducted in the United States (n=6), Canada (n=3), the United Kingdom (n=2), and Germany (n=2). There were 11 quantitative studies [11,30-34,36-38,40], 4 qualitative studies [27,35,39], and 3 mixed methods studies [12,29,41,42]. Some studies reported singular app domains while others reported multiple. Of the 18 studies included in the review, 13 reported on time to app abandonment [11,29-38,40,42], and one-third (n=6) described reasons for abandonment [12,27-29,39,41], with 1 study exploring both [11]. A full summary of study characteristics can be found in Table 2.

**Table 2:** Summary of characteristics of studies included in the scoping review (n=18).

Study characteristic		Studies, n (%)
**Year**		
	2019-2022	12 (69)
	2015-2018	5 (28)
	2011-2014	1 (6)
	2007-2010	—^a^
**Country**		
	United States	5 (28)
	Canada	3 (17)
	United Kingdom	2 (11)
	Germany	2 (11)
	Other	6 (36)
**Health domain**		
	Mental health	4 (22)
	Physical activity	3 (17)
	Diet	3 (17)
	Physical activity and diet	3 (17)
	Alcohol	2 (11)
	Diet and physical activity and mindfulness	1 (6)
	Alcohol consumption	1 (6)
	Sport and fitness	1 (6)
**Study design**		
	Quantitative	11 (61)
	Qualitative	4 (22)
	Mixed methods	3 (17)
**Outcome category**		
	Time to abandonment	13 (72)
	Reasons for abandonment	5 (28)
	Both	1 (6)

^a^Not applicable.

### Critical Appraisal Within Sources of Evidence

Thirteen studies [11,29-34,36-38,40-42] were assessed using the STROBE tool, with scores ranging from 16-21 out of a possible 26 (refer to Multimedia Appendix 4 for full results). All studies satisfied the criteria for having a structured abstracted summary, study rationale, stating key objectives and design elements, defining outcome variables, reporting demographic data, and setting information. Criteria less likely to be satisfied included indicating the study design in the title, rationale for the study size, any efforts to address bias, explanation of how missing data was handled, reasons for nonparticipation, and follow-up times (if relevant).

Five studies [12,27,28,35,39] were assessed using the COREQ tool, with scores ranging from 10-18 out of a possible 25 (refer to Multimedia Appendix 5 for full results). All studies satisfied the criteria for having a structured abstract summary, and study rationale, stating key objectives, design elements, definition of outcome measures, and participant characteristics. Criteria less likely to be satisfied included participant knowledge about the interviewers, evidence of interview pilot testing, presence of nonparticipants, details of when field notes were made, return of transcripts for correction, discussion of data saturation, participant feedback on findings, and duration of interviews.

The key findings table for the included studies can be found in Multimedia Appendix 6. Definitions of abandonment varied widely across the studies. For example, terminology included various terms such as abandonment, churn, and disengagement as well as retention. One study defined abandonment as no use for at least 7 days [31], while others defined it as no use for at least 14 days [33] and at least 4 weeks [40]. In addition, abandonment was measured at different time points across studies.

### Duration of App Use

**Figure 2:**
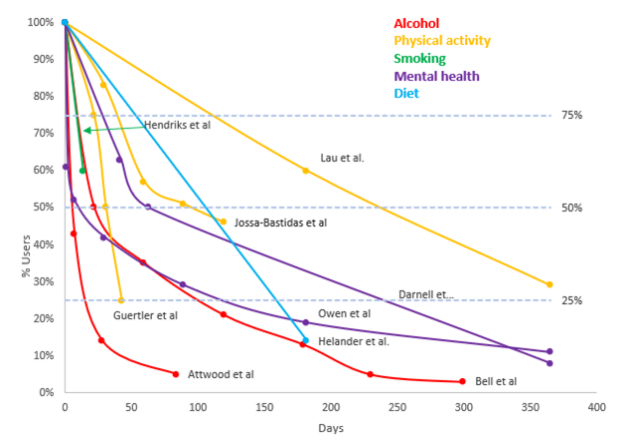
Duration of app use before abandonment. Four studies could not be included in the graph due to the way the data were reported.

Figure 2 graphically represents the duration apps were used before their abandonment across the examined studies. A curvilinear relationship can be seen, where health apps tend to be abandoned by many soon after they are acquired, with the rate of abandonment slowing with time. Though this general pattern is consistent across studies, the abandonment rate differed notably among studies. For example, Attwood et al [29] and Owen et al [42] found that half of the users stopped using the app within the initial days. In contrast, Lau et al [38] observed that users took approximately 240 days before half of them abandoned the app. Most studies (8 out of 10, assuming the studies which followed their users for <100 days continued on the same trajectory) reported that 50% or more of users had abandoned their app within the first 100 days of initial use, with a median abandonment rate at 100 days of approximately 70%.

While abandonment increased with time, no study reported complete abandonment by its participants. Attwood et al [29] and Bell et al [31] reported that over 95% of users had ceased using their apps by their last recorded follow-up timepoint, ranging from 80 to 300 days post initiation. Visual inspection suggested that app abandonment may occur more quickly for alcohol and smoking apps, whereas physical activity and possibly mental health apps appeared to achieve more prolonged use before abandonment.

### Reasons for Abandonment

In total, 6 studies collected data on reasons for app abandonment [11,12,27,28,39,41]. Table 3 summarizes the reasons for abandonment. A total of 22 unique reasons were identified, which were arranged into 12 subcategories and 6 overarching abandonment categories.

**Table 3:** Reasons for app abandonment were identified in the scoping review.

Category and subcategory		Codes
**Technical and functional issues**		
	Device compatibility	It no longer works on my phone [11]
	Data management	Data loss [27] Needing to create an account before using the app [27]
**Privacy**		
	General privacy	Privacy [27,41]
	Data sharing discomfort	Did not like sharing data with the app [11]
**User experience**		
	Effectiveness and engagement	Poor app effectiveness [11] The app was not engaging [41]
	Design and usability challenges	Confusing or difficult [11,28,39,41] Annoying notifications [28]
**Content and features**		
	Lacking features	Lack of personalization [28] Lack of accountability [28] Did not have the desired features [41] Lack of variety or options [27,39]
	Lacking content	Lack of knowledge within the app [12] Lack of “professionals” [12]
**Time and financial costs**		
	Unexpected expenses and obligations	Time to enter data [11,28] Hidden costs [11]
**Evolving user needs and goals**		
	Achievement and transition	No need to track anymore [12] I achieved my fitness goal [11,12,41]
	Interest and motivation shift	Fading motivation [11,12,41] Got bored [11,41]
	App selection and replacement	Identified the most suitable app and uninstalled the rest [11,41]

Technical and functional issues led to abandonment across mobile health apps [11,27]. For example, some users reported abandoning apps due to phone compatibility issues [11], while others described abandoning an app after a technical error and resultant data loss, and due to the need to create an account before using an app [27].

Concerns regarding privacy [27,41] along with user discomfort with and concern regarding data sharing [11] were contributing factors to mobile health app abandonment.

Poor user experience played a role in users abandoning mobile health apps [11,12,27,28,39,41]. Users reported that their apps were not engaging [41] or were perceived to lack effectiveness [11]. In addition, design and usability concerns contributed to abandonment, particularly where the apps were considered confusing to use [11,28,39,41]. In addition, annoyance from notifications [28] contributed to abandonment.

Participants reported lack of desired content and features contributed to them abandoning their apps. This included a lack of personalization, accountability [28], and other desired features [41], lacking variety [27,39]^,^ insufficient knowledge content [12], and a lack of professional advice from experts [12].

Time and financial costs also contributed to abandonment. This was particularly the case where there were unexpected hidden costs within apps [11] or burdensome data entry [11,28].

Finally, many users cited their evolving goals and needs as reasons for abandoning mobile health apps [11,12,41]. Entering a state of transition where users no longer needed to track their data [12] or had achieved their goals [11,12,41] led to user abandonment. Moreover, loss of motivation [11,12,41] and users reporting feeling bored with their app [11,41] resulted in mobile health app abandonment. Finally, some users reported downloading several potential apps, identifying the most suitable, and uninstalling the rest [11,41].

## Discussion

### Principal Findings

This study set out to examine the abandonment of health apps for lifestyle management in free-living conditions and to understand the underlying reasons for this abandonment. The duration of app use before abandonment varied across studies, but a common trend showed many users abandoning apps soon after the acquisition, with the abandonment rate then slowing over time. Most studies (7/9) reported more than 50% of users abandoning their apps within the first 100 days. The review identified several reasons for app abandonment, categorized into 6 overarching categories, that are technical and functional issues, privacy concerns, poor user experience, content and features, time and financial costs, and evolving user needs and goals. It appeared that physical activity and mental health apps might be used longer while smoking and alcohol apps appear to be abandoned more swiftly. Furthermore, our review highlights a considerable methodological diversity across studies, particularly in the measurement and terminology used to describe app abandonment.

Our findings reveal a common trend where users tend to abandon health apps soon after acquisition, with a slower rate of abandonment among the remaining users over time. The abandonment rates for different app domains varied, with physical activity apps (n=3) showing a 54%-75% abandonment rate, alcohol apps (n=2) at 95%-97%, smoking apps (n=1) at 40%, mental health apps (n=2) at 89%-92%, and diet apps (n=1) at 86%. These rates are notably higher than the 43% attrition rate reported in the meta-analysis by Meyerowitz-Katz et al [17], likely due to their inclusion of both controlled trial and free-living data. Controlled trials typically feature rigorous screening and follow-up processes that help reduce attrition. Conversely, our review found that in the majority of studies, over half of the users discontinued app use within the first 100 days, with a median abandonment rate of approximately 70%. This is consistent with industry reports indicating that around 69% of fitness and nutrition apps are abandoned within the first 90 days, followed by a slower rate of abandonment resulting in 81% abandonment over a year [9].

Our synthesis of qualitative data suggested that there are many and wide-ranging potential reasons for app abandonment. We identified 22 different reasons, which fell into 6 overarching categories, that are technical and functional issues, privacy concerns, poor user experience, content and features, time and financial costs, and evolving user needs and goals. Some of these findings inversely mirror the factors identified in the Jakob et al [16] and Amagai et al [14] reviews as influencing positive engagement with health apps. For instance, while Jakob et al [16] found that personalization enhances adherence, our study noted that a lack of personalization contributes to abandonment. Similarly, support from health professionals was seen as beneficial in Amagai and colleagues’ [14] study, whereas our findings indicate that the absence of expert content can lead to app discontinuation. This pattern is also observed with technical stability, where Jakob et al [16] identified it as a factor for adherence, contrasting with our identification of technical issues as a reason for abandonment. User experience issues such as being difficult or confusing to use were most consistently identified as being crucial to app abandonment in our review. This finding aligns with the results of a previous study, which extracted over 5 million user reviews from 278 apps and found that most complaints were linked to user experience issues [43].

The reasons for abandonment may be viewed in the context of the curvilinear relationship between app abandonment and time, suggesting there may be 2 key periods of abandonment, that consist of an initial phase characterized by early failure or disappointment, followed by a more gradual decline in interest. Early abandonment might be attributed to immediate deterrents such as device incompatibility, technical errors, or barriers like mandatory account creation before app usage. For example, research examining app user reviews found that complaints related to the sign-up process, such as complex sign-up forms and not receiving verification codes, were not uncommon [43]. These could potentially cause users to abandon apps before they have even begun using them. In contrast, factors like annoying notifications, a lack of desired features, or evolving user motivations could contribute to a more gradual disengagement over time. Research suggests that examining user experience over time is important to identify critical issues at different stages of usage [44].

Our findings suggested that the time to abandon mobile health apps may vary for different health app domains, with a seemingly sharper rate of abandonment for apps targeting smoking and alcohol, and more gradual abandonment for apps targeting physical activity and mental health. However, this finding should be interpreted with caution, as it is derived from visual inspection of data from a relatively small number of studies. The Meyerowitz-Katz et al [17] and Jakob et al [16] reviews each attempted to consider whether attrition and adherence (respectively) were associated with app domains and suggested that findings were inconclusive due to rates of attrition and adherence varying widely between different studies and methodological heterogeneity. Certainly, there are logical reasons for why app abandonment may vary by health domain. For example, smoking and alcohol apps target addictive behaviors, which can be particularly challenging to change [45,46]. Users might download these apps during a moment of motivation but find it difficult to maintain the commitment, leading to early abandonment. Also, the immediate withdrawal symptoms or cravings associated with quitting smoking or alcohol may lead to relapse and subsequent app abandonment. Other factors, like social features, stigma, and privacy concerns are also likely to differ between different health domains. For example, physical activity apps often include social features like sharing progress with friends or participating in challenges, which might enhance user engagement [47]. Conversely, there may be greater privacy concerns or stigma associated with using apps for smoking or alcohol cessation, particularly when using social support features [48,49].

Key strengths of our study include its comprehensive scope, covering a broad range of health app domains and contexts, to provide a holistic understanding of app abandonment. The adherence to the PRISMA-ScR protocol ensured a systematic and transparent review process, enhancing the reliability of our findings. We included methodological features that went beyond those expected for scoping reviews, including undertaking study screening in duplicate. The use of an inductive approach to content analysis allowed for the organization of categories directly from the data, offering insights that are grounded in empirical evidence.

The study’s most important limitations arose from the limitations of the evidence base itself, in particular, the heterogeneity in included studies’ methods and their definitions of abandonment, making comparison across studies challenging. Focusing on peer-reviewed journal articles and excluding gray literature might have introduced publication bias, as studies with significant or positive findings are more likely to be published. Limiting the review to English-language studies may exclude relevant research published in other languages, potentially introducing language bias. We also note that the use of negatively framed terminology to describe app abandonment (eg, abandonment, disengagement) might have resulted in the exclusion of studies that described how long participants used an app with only positively-framed terminology (eg, usage). Furthermore, while inductive content analysis is a strength, it also carries the risk of subjective interpretation of qualitative data.

This study highlights that to date, there has been limited research focused on app abandonment, an area that is crucial to optimizing the health benefits of apps. More longitudinal studies are needed to understand app abandonment patterns across various health behaviors. Furthermore, our field needs to establish a consistent definition and reporting approach to app abandonment within the literature to enable comparative analysis across studies.

### Conclusion

In conclusion, this study sheds light on the often-overlooked issue of health app abandonment in real-world settings, revealing key patterns and underlying reasons for this phenomenon. Our findings indicate that a large number of users abandon health apps shortly after downloading. The reasons for abandonment are multifaceted, encompassing technical and functional issues, privacy concerns, poor user experience, content inadequacies, financial considerations, and evolving user needs. These insights highlight critical areas for future research and development in health app design and implementation. There is a pressing need for more longitudinal studies to understand app abandonment patterns across various health behaviors and to establish a consistent definition and reporting approach for app abandonment. Such efforts are essential for optimizing the health-enhancing potential of health apps, guiding future development, and ensuring these digital tools effectively contribute to public health and individual well-being.

## Appendix

Multimedia Appendix 1 Preferred Reporting Items for Systematic reviews and Meta-Analyses extension for Scoping Reviews (PRISMA-ScR) Checklist.

Multimedia Appendix 2 Search strategy.

Multimedia Appendix 3 Data charting form.

Multimedia Appendix 4 STROBE (Strengthening the Reporting of Observational Studies in Epidemiology) Risk of bias results.

Multimedia Appendix 5 COREQ (Consolidated Criteria for Reporting Qualitative Research) Risk of bias results.

Multimedia Appendix 6 Study characteristics and key findings.

## Abbreviations

COREQ: Consolidated Criteria for Reporting Qualitative Research
MeSH: Medical Subject Headings
PRISMA: Preferred Reporting Items for Systematic Reviews and Meta-Analyses
PRISMA-ScR: Preferred Reporting Items for Systematic reviews and Meta-Analyses extension for Scoping Reviews
SPIDER: Sample, Phenomenon of Interest, Design, Evaluation, Research type
STROBE: Strengthening the Reporting of Observational Studies in Epidemiology
WHO: World Health Organization
