# Supplementary material for: When and Why Adults Abandon Lifestyle Behavior and Mental Health Mobile Apps: Scoping Review
Source: J Med Internet Res. 2024 Dec 18;26:e56897. doi: 10.2196/56897 (PMC11694054; doi:10.2196/56897)
Supplement: Multimedia Appendix 2 [file jmir_v26i1e56897_app2.docx]

# Multimedia Appendix 2. Search strategy

***Medline (353 results)***

| **#** | **Searches** | **Results** |
| --- | --- | --- |
| 1 | Cell phones/ | 9,835 |
| 2 | Smartphone/ | 8,465 |
| 3 | Mobile Applications/ | 10,775 |
| 4 | ("smart phone*" or smartphone* or "smart-phone*" or "cell*phone" or "mobile phone*" or "mobile-phone" or "mobile device" or "mobile telephone*" or i*Phone or IOS or "mobile health" or mhealth or "m-health" or apps or app or "mobile application*") | 83,369 |
| 5 | 1 or 2 or 3 or 4 | 86,451 |
| 6 | Exercise/ | 136,623 |
| 7 | Weight Loss/ | 42,471 |
| 8 | Sedentary Behavior/ | 13,146 |
| 9 | Diet/ | 181,359 |
| 10 | Mental Health/ | 56,975 |
| 11 | Alcohol Drinking/ | 74,092 |
| 12 | Smoking/ | 147,496 |
| 13 | Sleep/ | 65,150 |
| 14 | (Exercise* or sedentar* or sitting or inactiv* or "physical activit*" or "active living" or walk* or "active transport" or fitness or "weight loss" or "weight reduction" or "weight maintenance" or "maintaining weight" or "weight management" or diet* or nutrition or sleep or "mental health" or anxiety or depression or smoking or alcohol) | 3,531,772 |
| 15 | 6 or 7 or 8 or 9 or 10 or 11 or 12 or 13 or 14 | 3,531,772 |
| 16 | (abandon* or dropout or "drop out" or "non-use" or cease or ceasing or stop or disengag* or "stop using" or "quit using" or quit or "dis-engage" or "non-adher*" or "noncompli*" or "non-compli*" or "nonadher*" or "attrition" or "non-usage" or "nonusage") | 199,090 |
| 17 | 5 and 15 and 16 | 1,053 |
| 18 | limit 17 to (english language and humans and yr="2007 -Current") | 683 |
| 19 | ("RCT" or "clinical trial*" or "controlled study" or "randomized controlled trial*" or "randomised controlled trial*") | 1,627,464 |
| 20 | 18 not 19 | 353 |

***Embase (489 results)***

| **#** | **Searches** | **Results** |
| --- | --- | --- |
| 1 | Cell phones/ | 19,264 |
| 2 | Smartphone/ | 22,853 |
| 3 | Mobile Applications/ | 18,268 |
| 4 | ("smart phone*" or smartphone* or "smart-phone*" or "cell*phone" or "mobile phone*" or "mobile-phone" or "mobile device" or "mobile telephone*" or i*Phone or IOS or "mobile health" or mhealth or "m-health" or apps or app or "mobile application*") | 130,072 |
| 5 | 1 or 2 or 3 or 4 | 130,073 |
| 6 | Exercise/ | 352,177 |
| 7 | Weight Loss/ | 58,014 |
| 8 | Sedentary Behavior/ | 8,536 |
| 9 | Diet/ | 287,513 |
| 10 | sleep/ | 120,617 |
| 11 | drinking behavior/ | 56,522 |
| 12 | mental health/ | 192,847 |
| 13 | smoking/ | 369,547 |
| 14 | (Exercise* or sedentar* or sitting or inactiv* or "physical activit*" or "active living" or walk* or "active transport" or fitness or "weight loss" or "weight reduction" or "weight maintenance" or "maintaining weight" or "weight management" or diet* or nutrition or sleep or alcohol or smoking or "mental health" or anxiety or depression) | 5,460,552 |
| 15 | 6 or 7 or 8 or 9 or 10 or 11 or 12 or 13 or 14 | 5,467,203 |
| 16 | (abandon* or dropout or "drop out" or "non-use" or cease or ceasing or stop or disengag* or "stop using" or "quit using" or quit or "dis-engage" or "non-adher*" or "noncompli*" or "non-compli*" or "nonadher*" or "attrition" or "non-usage" or "nonusage") | 302,505 |
| 17 | 5 and 15 and 16 | 1,377 |
| 18 | limit 17 to (human and english language and yr="2007 -Current") | 1,308 |
| 19 | ("RCT" or "clinical trial*" or "controlled study" or "randomized controlled trial*" or "randomised controlled trial*") | 10,557,444 |
| 20 | 18 not 19 | 489 |

***PsycINFO (263 results)***

| **#** | **Searches** | **Results** |
| --- | --- | --- |
| 1 | Mobile Phones/ | 5,281 |
| 2 | Smartphones/ | 2,850 |
| 3 | mobile application/ | 2,050 |
| 4 | ("smart phone*" or smartphone* or "smart-phone*" or "cell*phone" or "mobile phone*" or "mobile-phone" or "mobile device" or "mobile telephone*" or i*Phone or IOS or "mobile health" or mhealth or "m-health" or apps or app or "mobile application*") | 24,574 |
| 5 | 1 or 2 or 3 or 4 | 24,574 |
| 6 | Exercise/ | 26,600 |
| 7 | Weight Loss/ | 4,415 |
| 8 | Sedentary Behavior/ | 2,287 |
| 9 | Diets/ | 14,900 |
| 10 | Sleep/ | 27,683 |
| 11 | Mental Health/ | 83,250 |
| 12 | Drinking Behavior/ | 2,000 |
| 13 | Tobacco Smoking/ | 35,078 |
| 14 | (Exercise* or sedentar* or sitting or inactiv* or "physical activit*" or "active living" or walk* or "active transport" or fitness or "weight loss" or "weight reduction" or "weight maintenance" or "maintaining weight" or "weight management" or diet* or nutrition or sleep or alcohol or smoking or "mental health" or anxiety or depression) | 1,130,761 |
| 15 | 6 or 7 or 8 or 9 or 10 or 11 or 12 or 13 or 14 | 1,131,118 |
| 16 | (abandon* or dropout or "drop out" or "non-use" or cease or ceasing or stop or disengag* or "stop using" or "quit using" or quit or "dis-engage" or "non-adher*" or "noncompli*" or "non-compli*" or "nonadher*" or "attrition" or "non-usage" or "nonusage") | 84,305 |
| 17 | 5 and 15 and 16 | 448 |
| 18 | limit 17 to (peer reviewed journal and human and english language and yr="2007 -Current") | 376 |
| 19 | ("RCT" or "clinical trial*" or "controlled study" or "randomized controlled trial*" or "randomised controlled trial*") | 98,818 |
| 20 | 18 not 19 | 263 |

***Scopus (700 results)***

| ***#*** | ***Searches*** |
| --- | --- |
| 1 | "smart phone*" OR smart-phone* OR "cell*phone*" OR "cell-phone*" OR "mobile phone*" OR "mobile device" OR "mobile telephone*" OR i*phone* OR android* OR ios OR "mobile health" OR "mhealth" OR "m-health" OR app OR apps OR "mobile application*" |
| 2 | exercise* OR sedentary* OR sitting OR inactive* OR "physical activit*" OR "active living" OR walk* OR "active transport*" OR fitness OR "weight loss" OR "weight reduction" OR "weight maintenance" OR "maintaining weight" OR "weight management" OR diet* OR nutrition OR sleep OR alcohol OR smoking OR "mental health" OR anxiety OR depression |
| 3 | abandon* OR dropout OR "drop out" OR "nonusage" OR "non-use" OR cease OR ceasing OR stop OR disengage* OR "stop using" OR "quit using" OR quit OR "dis-engage*" OR "non-adhere*" OR "noncompli*" OR "nonadhere*" OR "attrition" OR "non-usage" |
| 4 | LIMIT-TO ( SRCTYPE , "j" ) ) AND ( LIMIT-TO ( PUBYEAR , 2023 ) OR LIMIT-TO ( PUBYEAR , 2022 ) OR LIMIT-TO ( PUBYEAR , 2021 ) OR LIMIT-TO ( PUBYEAR , 2020 ) OR LIMIT-TO ( PUBYEAR , 2019 ) OR LIMIT-TO ( PUBYEAR , 2018 ) OR LIMIT-TO ( PUBYEAR , 2017 ) OR LIMIT-TO ( PUBYEAR , 2016 ) OR LIMIT-TO ( PUBYEAR , 2015 ) OR LIMIT-TO ( PUBYEAR , 2014 ) OR LIMIT-TO ( PUBYEAR , 2013 ) OR LIMIT-TO ( PUBYEAR , 2012 ) OR LIMIT-TO ( PUBYEAR , 2011 ) OR LIMIT-TO ( PUBYEAR , 2010 ) OR LIMIT-TO ( PUBYEAR , 2009 ) OR LIMIT-TO ( PUBYEAR , 2008 ) OR LIMIT-TO ( PUBYEAR , 2007 ) ) AND ( LIMIT-TO ( LANGUAGE , "english" |
| 5 | "controlled study" ) OR EXCLUDE ( EXACTKEYWORD , "randomized controlled trial" ) OR EXCLUDE ( EXACTKEYWORD , "randomized controlled trial (topic)" ) OR EXCLUDE ( EXACTKEYWORD , "clinical trial" |
