# Supplementary material for: When and Why Adults Abandon Lifestyle Behavior and Mental Health Mobile Apps: Scoping Review
Source: J Med Internet Res. 2024 Dec 18;26:e56897. doi: 10.2196/56897 (PMC11694054; doi:10.2196/56897)
Supplement: Multimedia Appendix 4 [file jmir_v26i1e56897_app4.docx]

Multimedia Appendix 4. STROBE Risk of bias results

| **Criteria** | Attwood et al. 2017 | Bell et al. 2020 | Darnell et al. 2022 | Giraldo‐O'Meara & Doron 2021 | Guertler et al. 2015 | Helander et al. 2014 | Jossa-Bastidas et al. 2021 | König et al. 2018 | Krebs & Duncan 2015 | Kwon et al. 2021 | Lau et al. 2022 | Mustafa et al. 2022 | Owen et al. 2015 |
| --- | --- | --- | --- | --- | --- | --- | --- | --- | --- | --- | --- | --- | --- |
| **Title and Abstract** |  |  |  |  |  |  |  |  |  |  |  |  |  |
| Indicate the study design within the title | 1 | 1 | 1 | 0 | 0 | 1 | 0 | 0 | 1 | 1 | 0 | 1 | 0 |
| Provide an abstract summary | 1 | 1 | 1 | 1 | 1 | 1 | 1 | 1 | 1 | 1 | 1 | 1 | 1 |
| **Introduction** |  |  |  |  |  |  |  |  |  |  |  |  |  |
| Describe the scientific background and rationale for the study | 1 | 1 | 1 | 1 | 1 | 1 | 1 | 1 | 1 | 1 | 1 | 1 | 1 |
| State the specific objectives | 1 | 1 | 1 | 1 | 1 | 1 | 1 | 1 | 1 | 1 | 1 | 1 | 1 |
| **Methods** |  |  |  |  |  |  |  |  |  |  |  |  |  |
| Present key elements of study design early in the paper | 1 | 1 | 1 | 1 | 1 | 1 | 1 | 1 | 1 | 1 | 1 | 1 | 1 |
| Describe the setting, locations, and relevant dates, including periods of recruitment, exposure, follow‐up, and data collection | 1 | 1 | 1 | 1 | 1 | 1 | 1 | 1 | 1 | 1 | 1 | 1 | 1 |
| Give the eligibility criteria, and the sources and methods of selection of participants/case selection. *Cohort study* Describe methods of follow‐up. | 1 | 1 | 1 | 1 | 1 | 1 | 1 | 1 | 1 | 1 | 1 | 1 | 1 |
| Clearly define all outcomes, exposures, predictors, potential confounders, and effect modifiers. Give diagnostic criteria, if applicable | 1 | 1 | 1 | 1 | 1 | 1 | 1 | 1 | 1 | 1 | 1 | 1 | 1 |
| For each variable of interest, give sources of data and details of methods of assessment (measurement). Describe comparability of assessment methods more than one group | 1 | 1 | 1 | 1 | 1 | 1 | 1 | 1 | 1 | 1 | 1 | 1 | 1 |
| Describe any efforts to address potential sources of bias | 0 | 1 | 0 | 0 | 0 | 0 | 0 | 0 | 1 | 0 | 1 | 1 | 0 |
| Explain how the study size was arrived at | 1 | 0 | 0 | 0 | 0 | 0 | 0 | 0 | 0 | 0 | 0 | 0 | 0 |
| Explain how quantitative variables were handled in the analyses. If applicable, describe which groupings were chosen and why | 1 | 1 | 1 | 1 | 1 | 1 | 1 | 1 | 1 | 1 | 1 | 1 | 1 |
| Describe all statistical methods, including those used to control for confounding, and any methods used to examine subgroups and interactions, and any sensitivity analyses | 1 | 1 | 1 | 1 | 1 | 1 | 1 | 1 | 1 | 1 | 1 | 1 | 1 |
| Explain how missing data was addressed | 0 | 0 | 1 | 0 | 0 | 0 | 0 | 1 | 0 | 0 | 1 | 0 | 0 |
| **Results** |  |  |  |  |  |  |  |  |  |  |  |  |  |
| Report numbers of individuals at each stage of study (e.g., numbers potentially eligible, examined for eligibility, confirmed eligible, included in the study, completing follow‐up, and analysed) | 1 | 1 | 1 | 1 | 1 | 1 | 1 | 1 | 1 | 1 | 1 | 1 | 1 |
| Give reasons for non‐participation at each stage, with the use of a flow diagram | 1 | 0 | 0 | 0 | 0 | 0 | 0 | 1 | 0 | 0 | 0 | 0 | 0 |
| Give characteristics of study participants (e.g., demographic, clinical, social) and information on exposures and potential confounders | 1 | 1 | 1 | 1 | 1 | 1 | 1 | 1 | 1 | 1 | 1 | 1 | 1 |
| Indicate number of participants with missing data for each variable of interest | 0 | 0 | 1 | 0 | 0 | 0 | 0 | 1 | 0 | 0 | 0 | 0 | 0 |
| *Cohort Study* Summarise follow‐up time (e.g., average and total amount) | 0 | 0 | 0 | 0 | 0 | 0 | 0 | 0 | 0 | 0 | 0 | 0 | 0 |
| *Cohort study* Report numbers of outcome events or summary measures over time; *Cross‐sectional study* Report numbers of outcome events or summary measures | 1 | 1 | 1 | 1 | 1 | 1 | 1 | 1 | 1 | 1 | 1 | 1 | 1 |
| Give unadjusted estimates and, if applicable, confounder‐adjusted estimates and their precision (e.g., 95% confidence interval). Make clear which confounders were adjusted for and why they were included. | 0 | 1 | 1 | 0 | 1 | 0 | 0 | 0 | 1 | 0 | 1 | 0 | 0 |
| Report category boundaries when continuous variables were categorized | 0 | 1 | 0 | 0 | 1 | 1 | 1 | 0 | 1 | 0 | 1 | 0 | 0 |
| **Discussion** |  |  |  |  |  |  |  |  |  |  |  |  |  |
| Summarise key results with reference to study objectives | 1 | 1 | 1 | 1 | 1 | 1 | 1 | 1 | 1 | 1 | 1 | 1 | 1 |
| Discuss limitations of the study, taking into account sources of potential bias or imprecision, with mention of both direction and magnitude of potential bias | 1 | 1 | 1 | 1 | 1 | 1 | 0 | 1 | 1 | 1 | 1 | 1 | 1 |
| Give a cautious overall interpretation of results considering objectives, limitations, multiplicity of analyses, results from similar studies, and other relevant evidence. Include discussion of the generalisability (external validity) of the study results | 1 | 1 | 1 | 1 | 1 | 1 | 0 | 1 | 1 | 1 | 1 | 1 | 1 |
| **Funding** |  |  |  |  |  |  |  |  |  |  |  |  |  |
| Give the source of funding and the role of the funders for the present study and, if applicable, for the original study on which the present article is based | 1 | 1 | 1 | 0 | 1 | 1 | 1 | 1 | 1 | 1 | 1 | 1 | 0 |
| **Number of criteria satisfied (/26)** | 20 | 21 | 21 | 16 | 19 | 19 | 16 | 20 | 21 | 18 | 21 | 19 | 16 |
