# Supplementary material for: When and Why Adults Abandon Lifestyle Behavior and Mental Health Mobile Apps: Scoping Review
Source: J Med Internet Res. 2024 Dec 18;26:e56897. doi: 10.2196/56897 (PMC11694054; doi:10.2196/56897)
Supplement: Multimedia Appendix 5 [file jmir_v26i1e56897_app5.docx]

Multimedia Appendix 5. COREQ Risk of bias results

| **Criteria** | Alqahtani & Orji 2020 | Hendriks et al. 2020 | Lupton 2020 | Lu et al. 2021 | Vaghefi & Tulu 2019 |
| --- | --- | --- | --- | --- | --- |
| **Research Team and Reflexivity** |  |  |  |  |  |
| *Personal Characteristics* |  |  |  |  |  |
| Information regarding who conducted the interviews or focus groups, credentials of the researchers, their occupation and experience levels | 0 | 1 | 1 | 1 | 1 |
| *Relationship with Participants* |  |  |  |  |  |
| Declaration of any relationship established before the study/participant knowledge about the interviewers | 0 | 0 | 0 | 0 | 0 |
| Characteristics of the interviewer/s | 0 | 1 | 0 | 1 | 1 |
| **Study Design** |  |  |  |  |  |
| *Theoretical Framework* |  |  |  |  |  |
| Methodological orientation underpinning the study | 1 | 1 | 1 | 1 | 1 |
| *Participant Selection* |  |  |  |  |  |
| Description of how the participants were selected and methods used to approach | 1 | 1 | 1 | 1 | 1 |
| Sample size of the study is reported | 1 | 1 | 1 | 1 | 1 |
| People who refused to participate or dropped out the study; with reasons | 1 | 1 | 0 | 0 | 1 |
| *Setting* |  |  |  |  |  |
| Location of data collection | 0 | 1 | 1 | 0 | 0 |
| Any presence of non‐participants during the interview process | 0 | 1 | 0 | 0 | 0 |
| Reporting of demographic and characteristic information of the participants | 0 | 0 | 1 | 1 | 1 |
| *Data Collection* |  |  |  |  |  |
| Questions used provided by the authors | 0 | 1 | 1 | 1 | 1 |
| Evidence of pilot testing of the interviews | 0 | 0 | 0 | 0 | 0 |
| Interview recording method (e.g., visual/audio) | 0 | 1 | 1 | 1 | 1 |
| Detail of when field notes were made | 0 | 0 | 0 | 0 | 0 |
| Duration of the interviews or focus group | 0 | 1 | 0 | 0 | 1 |
| Discussion of data saturation | 0 | 1 | 0 | 0 | 0 |
| Return of transcripts to participants for comment and/or correction | 0 | 0 | 0 | 0 | 0 |
| **Analysis & Findings** |  |  |  |  |  |
| *Data Analysis* |  |  |  |  |  |
| Number of data coders who coded the data | 1 | 1 | 1 | 1 | 1 |
| Description of the coding tree used | 1 | 0 | 1 | 1 | 1 |
| Clarification whether themes were identified in advance or derived from the data | 1 | 1 | 1 | 1 | 1 |
| Described software used to manage the data | 0 | 1 | 0 | 0 | 1 |
| Participant feedback on the findings | 0 | 0 | 0 | 0 | 0 |
| *Reporting* |  |  |  |  |  |
| Participant quotations presented to illustrate the themes/findings | 1 | 1 | 1 | 1 | 1 |
| Consistency between the data presented and the findings | 1 | 1 | 1 | 1 | 1 |
| Clear presentation of major themes in the findings, with description of diverse cases or discussion of minor themes as relevant | 1 | 1 | 1 | 1 | 1 |
| **Number of criteria satisfied (/25)** | **10** | **18** | **14** | **14** | **17** |
