# Supplementary material for: When and Why Adults Abandon Lifestyle Behavior and Mental Health Mobile Apps: Scoping Review
Source: J Med Internet Res. 2024 Dec 18;26:e56897. doi: 10.2196/56897 (PMC11694054; doi:10.2196/56897)
Supplement: Multimedia Appendix 6 [file jmir_v26i1e56897_app6.docx]

Multimedia Appendix 6. Study characteristics and key findings

| **Study** | **Health domain** | **Study design** | **Population** (n, % female, age range or mean age) | **Outcome measures** | **Key findings** |
| --- | --- | --- | --- | --- | --- |
| Alqahtani & Orji 2020 [27] | Mental health | Qualitative analysis of app reviews via App Store and Google Play | n=1,500, sex N/A, age N/A | Themes relating to user needs, experience, satisfaction, and expectations of mental health apps | Identified common complaints relating to data loss, privacy, and lack of variety/options |
| Attwood et al. 2017 [29] | Alcohol | Mixed methods | n=102,345, 59% female, age range=17-75+ years years | % retention (i.e., recorded alcohol consumption at least once during the week) after weeks 1, 4, and 12; reasons for app abandonment (data not available) | % retention at week 1: 43%, week 4: 14%, week 12 5% |
| Bell et al. 2020 [31] | Alcohol | Longitudinal observational study | n=19,233, 50% female, mean age=44 years | % abandonment (i.e., the first day of 7 or more consecutive days of no use) | % abandonment at 22 days: 50%, 60 days: 65%, 120 days: 79%, 180 days: 87%, 240 days: 95%, 300 days: 97% |
| Darnell et al. 2022 [40] | Mental health | Longitudinal observational study | n=5,890, 76% female, age range=18-50+ years | % disengagement (i.e., no client-initiated communication for more than 4 weeks) | % disengagement at week 6: 37%, week 9: 50%, week 52: 92% |
| Giraldo‐O'Meara & Doron 2021 [32] | Mental health | Longitudinal observational study | n=5,320, 25% female, mean age=27 years | % of participants that completed the Level 1 assessment 1 who went on to complete assessments at Levels 20 and 46 | Level 1 completion: n=5,320 (100%), Level 20: n=1,034 (19%), Level 46: n=165 (16%) |
| Guertler et al. 2015 [33] | Physical activity | Longitudinal observational study | n=1,451, 72% female, mean age=38 years | The number of days after which 25%, 50% and 75% of users had abandoned the app (ie. not logged physical activity data for at least 14 days) | Abandonment 25%: 22 days, 50%: 31 days, 75%: 43 days |
| Helander et al. 2014 [34] | Diet | Longitudinal observational study | n=189,770, sex N/A, age N/A | % of dropouts (i.e., users who took no valid pictures or only one valid picture), % who used the app for less than 7 days, % who used the app from more than 7 days | 163,949/189,770 (86%) were classed as dropouts. 131,001 (69%) took no valid pictures. 32,948 (17%) took one valid picture. 20,926 (11%) used the app for less than 7 days. 4,895 (2.58%) used the app from more than 7 days. |
| Hendriks et al. 2022 [35] | Smoking | Qualitative interviews | n=10, 60% female, mean age=41 years | Number of users reporting that they used their selected app after 2 weeks | 60% of participants had used their app after 2 weeks |
| Jossa-Bastidas et al. 2021 [30] | Physical activity | Longitudinal observational study | n=246, 50% female, mean age=40 years | Number of users who logged no exercise (average daily 0 seconds) across four monthly time points | Month 1: 41/246, 17%  Month 2: 106/246 43%,  Month 3: 121/206 49%,  Month 4: 132/206 54% |
| König et al. 2018 [36] | Diet & fitness | Longitudinal observational study | n=1,236, 64% female, mean age=41 years | % of participants who reported they had had never installed, previously installed, or currently installed a nutrition or fitness app. | Diet: 77% (806/1,051) never installed, 15% (159/1,051) previously installed, and 8% (86/1,051) currently installed.  Fitness: 52% (550/1,051) never installed, 23% (246/1,051) previously installed, and 24% (255/1,051) currently installed. |
| Krebs & Duncan 2015 [11] | Physical activity & diet | Cross-sectional survey | n=1,604, 50% female, mean age=40 years | % reporting they downloaded an app, % reporting they no longer use an app, % selecting reasons for app abandonment | 934/1,604 (58%) had downloaded a physical activity or diet app. 427/934 (46%) reported no longer using an app.  % selecting reasons for app abandonment: data entry burden (45%), loss of interest (41%), hidden costs (36%), confusing apps (33%), sharing data (29%), didn’t help me (19%), found better apps (16%), met my goals (10%), no longer works on my phone (10%), Other (3%) |
| Kwon et al. 2021 [37] | Physical activity & diet | Longitudinal observational study | n=1,868, 88% female, mean age=31 years | Mean number of days of service use in the “churn” group (i.e., users who received a refund after the 7 day trial period and before the paid 16-week program ended) | 44 days was the mean service use in the “churn” group |
| Lau et al. 2022 [38] | Physical activity | Longitudinal observational study | n=41,207, 67% female, mean age=35 | Total number of active weeks (mean daily step count > 0 for a week), % who had a break (i.e., period with zero steps for two or more consecutive weeks) | 60% used the app for 6 months. 29% used the app for 52 weeks. 69% had a “break”, with 42% of them not returning |
| Lu et al. 2021 [12] | Diet | Mixed methods | n=18, 61% female, mean age=25 years | Reasons for app abandonment | Identified reasons for app abandonment included not needing to track diet anymore, fading motivation, met diet goal, shallow app knowledge, not backed by “professionals” |
| Lupton 2020 [39] | Diet | Qualitative interviews | n=66, 100% female, age range=21-74 years | Reasons for app abandonment | Identified reasons for app abandonment included “Didn’t have the foods I eat” (lack of options) and “App is tedious to use” |
| Mustafa et al. 2022 [41] | Sport & fitness | Cross-sectional survey | n=209, 55% female, mean age=29 years | Reasons for app abandonment | Main reason for app abandonment: Identify the most suitable and uninstall the rest (21%), does not have the features I want (19%), get bored or lose motivation (16%), lose motivation to reach personal health goals (16%), app not engaging (10%), app not easy to use (9%), achieved fitness goal (6%), privacy (3%) |
| Owen et al. 2015 [42] | Mental health | Mixed methods (qualitative section was not relevant) | n=153,834; sex N/A, age N/A | % rolling retention (users whose last day of app use was some time after a given time point) | % retention at 1 day: 61%, 1 week: 52%, 1 month: 42%, 3 months: 29%, 6 months: 19%, 1 year: 11% |
| Vaghefi & Tulu 2019 [28] | Diet, physical activity & mindfulness | Qualitative longitudinal study | n=17, 70% female, age range=18-51 years | Reasons for app abandonment | Identified reasons for app abandonment included lack of accountability, annoying notifications, burden of data entry, navigation difficulty, depth of knowledge, and lack of personalisation |

Note: N/A = not available
